# The role of essential oils as eco-friendly strategy to control biofilm collected in the Colosseum (Rome, Italy)

**DOI:** 10.1007/s00253-025-13433-1

**Published:** 2025-02-18

**Authors:** Roberta Ranaldi, Lorenza Rugnini, Giada Migliore, Flavia Tasso, Francesco Gabriele, Nicoletta Spreti, Francesco Scuderi, Roberto Braglia, Patrick Di Martino, Angelica Pujia, Antonella Canini

**Affiliations:** 1 Department of Biology, Tor Vergata University of Rome, Via Della Ricerca Scientifica 1, 00133 Rome, Italy; 2 Department of Territorial and Production Systems Sustainability, ENEA, Via Anguillarese 301, 00123 Rome, Italy; 3 Department of Physical and Chemical Sciences, University of Aquila, Via Vetoio, Coppito, 67100 L’Aquila, Italy; 4 ERRMECe Laboratory, University of Cergy-Paris, Rue 13 Descartes Site de Neuville-Sur-Oise, 95031 Cergy-Pontoise, France; 5 Parco Archeologico del Colosseo, Piazza Santa Maria Nova 53, 00186 Rome, Italy

**Keywords:** Colosseum hypogeum, Biodeterioration, Biofilm, Microorganisms, Green biocides, Essential oils

## Abstract

**Abstract:**

The control of biodeteriogenic microorganisms is essential for the management of heritage sites. Many conventional biocides are no longer available because they have lost their efficacy or have been withdrawn from the market due to their danger to humans and the environment. Therefore, new effective and sustainable biocides are needed, such as plant extracts that could be a good alternative. In this study, essential oils (EOs) of *Ocimum basilicum* L., *Cinnamomum verum* Presl, *Lavandula angustifolia* Mill., *Origanum vulgare* L., *Thymus vulgaris* L. and *Melaleuca alternifolia* Maiden & Betche were tested as green biocides against microorganisms collected from biofilms in the hypogeum of the Colosseum (Rome, Italy). Biocidal screening was first carried out on phototrophic microorganisms grown on BG11 agar culture medium. The efficacy was assessed by measuring photosynthetic activity with a mini-PAM portable fluorometer, and by determining morphological changes or the absence of autofluorescence using light microscopy and confocal laser scanning microscopy. The most effective EOs against phototrophs were further tested to inhibit the growth of heterotrophic fungi and bacteria in order to identify those with a broad-spectrum action. The EOs of cinnamon, oregano and thyme at 5% concentration (*v/v*) were the most effective against the microorganisms isolated from the biofilms in the Colosseum. These EOs represent a green alternative to traditional chemical biocides due to their activity against a wide range of microorganisms and their complex composition which suggests the potential to reduce the risk of microbial resistance.

**Key points:**

- *Biofilms collected from the Colosseum hypogeum were characterized*
- *EOs tested as biocides against phototrophs and heterotrophs in Colosseum biofilms.*
- *Cinnamon, oregano, and thyme EOs show broad-spectrum action at 5% concentration.*

**Graphical Abstract:**

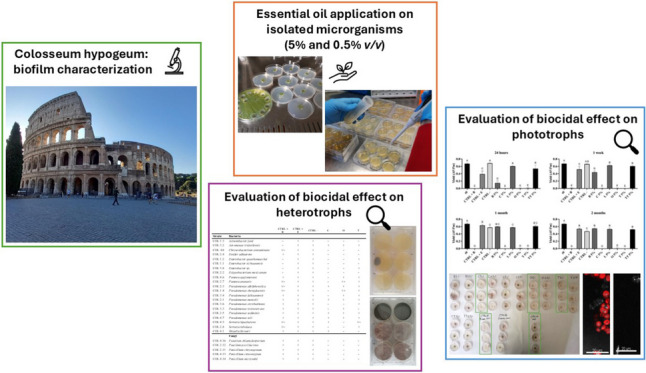

**Supplementary Information:**

The online version contains supplementary material available at 10.1007/s00253-025-13433-1.

## Introduction

Archaeological sites preserve evidence of the past and provide valuable knowledge for researchers, as well as attracting interest as tourist destinations. The colonization of these sites by plants and microorganisms has led to changes in the ecological dynamics, forming a relationship between the material construction and living organisms. In some cases, this interaction can enhance the cultural heritage, but in most cases, the biofilm that develops are responsible for biodeterioration rather than preservation (Carrari et al. 2022; Lisci et al. 2003). Cultural heritage biodeteriogens are complex communities, consisting of bacteria (including cyanobacteria), microalgae, fungi, lichens, mosses and plants whose composition is influenced by abiotic factors such as substrate bioreceptivity and environmental conditions (temperature, relative humidity and light exposure) (Chen et al. 2022). The control of these biodeteriogenic microorganisms is essential for the management of heritage sites, not only for their preservation. Traditional methods of remediation involve chemical treatments with biocides combined with physical methods. However, these methods can be toxic to operators and harmful to the environment and tend to have a short-lasting effect, contributing to the development of resistant species on cultural heritage surfaces (Cirone et al. 2023; Mazzoli et al. 2018). To address these limitations, researchers and conservators are exploring many innovative plant-based biocides, such as essential oils (EOs) or plant extracts, to provide eco-friendly alternatives for controlling biodeteriogens. Botanical extract and especially EOs are widely employed in the food industry as an alternative to synthetic preservatives, in pharmacology to combat multi-drug-resistant bacteria, and in cosmetology (Angane et al. 2022; de Sousa et al. 2023; Gismondi et al. 2020, 2021; Li et al. 2023; Pisanti et al. 2022), due to their antibacterial, antifungal, antiviral, insecticidal, antioxidant and enzyme inhibitory activities (Alvarez-Martínez et al. 2021; Mutlu-Ingok et al. 2020). Their specific mode of action is complex due to the large number of components, mainly terpenes. However, further investigation is required to understand their action, which is thought to involve different pathways acting on different targets (Andrade-Ochoa et al. 2021).

The hypogeum of the Colosseum (Rome, Italy) underwent between 2019 and 2021 a major conservation campaign, aiming at restoring and reopen to the public areas of the monument that have been closed for a long time. This restoration involved surfaces and structures of this area by intervening on brick walls, tufa and travertine blocks, cocciopesto and pozzolanic wall plaster as well as *opus spicatum* brick floors. Soon after its inauguration, the hypogeum was affected by the growth of biodeteriogens, mainly on the floors, but also on vertical surfaces due to the presence of a surface groundwater and drainage collection systems. Over the last 3 years, repeated use of commercial biocides based on quaternary ammonium salt has favoured the selection of a microbial community partially resistant to biocidal treatments. Routine site management involves regular disinfection cycles associated with water washing. Over time, the frequency of these interventions has increased, resulting in a great deal of effort for the restorers and increased stress on the materials. For several years now, “Parco archeologico del Colosseo”, within the framework of the “Parco Green” project (https://colosseo.it/parco-green/), has been pursuing a path of sustainability and green conversion of its activities, and with this in mind, has undertaken this experimentation aimed at limiting the use of traditional biocide products.

The microorganisms in the Colosseum hypogeum floor therefore represent a significant challenge for sustainable biocidal application due to their complexity and resistance to conventional treatments. In the present study, biofilm was collected from the Colosseum hypogeum and characterized by microscopic observations. In the laboratory, phototrophic and heterotrophic microorganisms were isolated, identified and cultured. The strains were then used as target species to test the biocidal efficacy of *Ocimum basilicum* L., *Cinnamomum verum* Presl, *Lavandula angustifolia* Mill., *Origanum vulgare* L., *Thymus vulgaris* L. and *Melaleuca alternifolia* Maiden & Betche. For phototrophs, the effect of the treatments was evaluated by measuring photosynthetic activity, determining morphological changes and the absence of autofluorescence. Then, the most effective EOs against phototrophs were tested to inhibit the growth of fungi and heterotrophic bacteria, to find those effective against a wide range of microorganisms in the biofilm and to provide an alternative solution to traditional treatments while reducing the microbial resistance.

## Materials and methods

### Biofilm sampling site

**Fig. 1:**
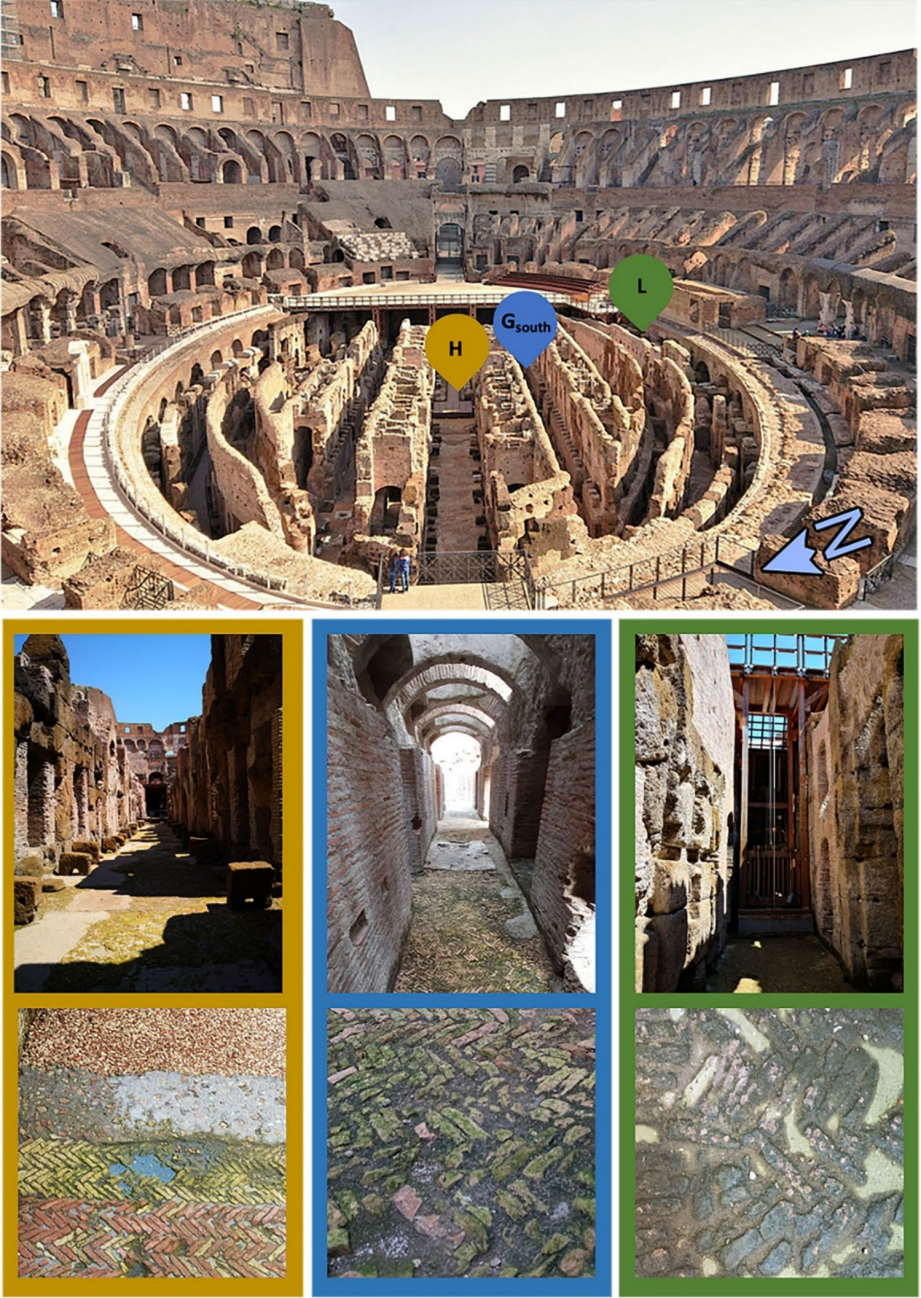
Selected areas in the Colosseum hypogeum: H (in yellow), G-south (in blue) and L (in green) sites are characterized by different environmental conditions and different construction materials and techniques, “cocciopesto” and “*opus spicatum*” floors

Three areas of the hypogeum of the Colosseum were selected based on the different environmental conditions, including solar radiation, exposure, and soil humidity, as well as the type of building material and construction techniques employed. These include the use of *opus spicatum* and modern “cocciopesto” floors which have distinct physical and chemical properties that influence their bioreceptivity (Fig. 1). The *opus spicatum* refers to a flooring technique characterized by the arrangement of small rectangular bricks in a herringbone pattern. In contrast, “cocciopesto” is a type of flooring made from a mixture of lime and finely crushed brick fragments, resulting in a durable and waterproof surface. The H site is located in the central corridor, exposed to direct light for most of the day and away from water accumulation points. It has a mixed floor of “cocciopesto” and *opus spicatum* floors. The G-south site is adjacent to the central corridor, partially covered by open sections, permanently shaded, away from drainage points and water accumulation, the floor is predominantly *opus spicatum*. The L site is at the end of the side corridor, not covered but in the shade, near the drains and the water reservoir under the floor of the ground. The floor is mainly “cocciopesto” with inclusions of *opus spicatum*.

**Fig. 2:**
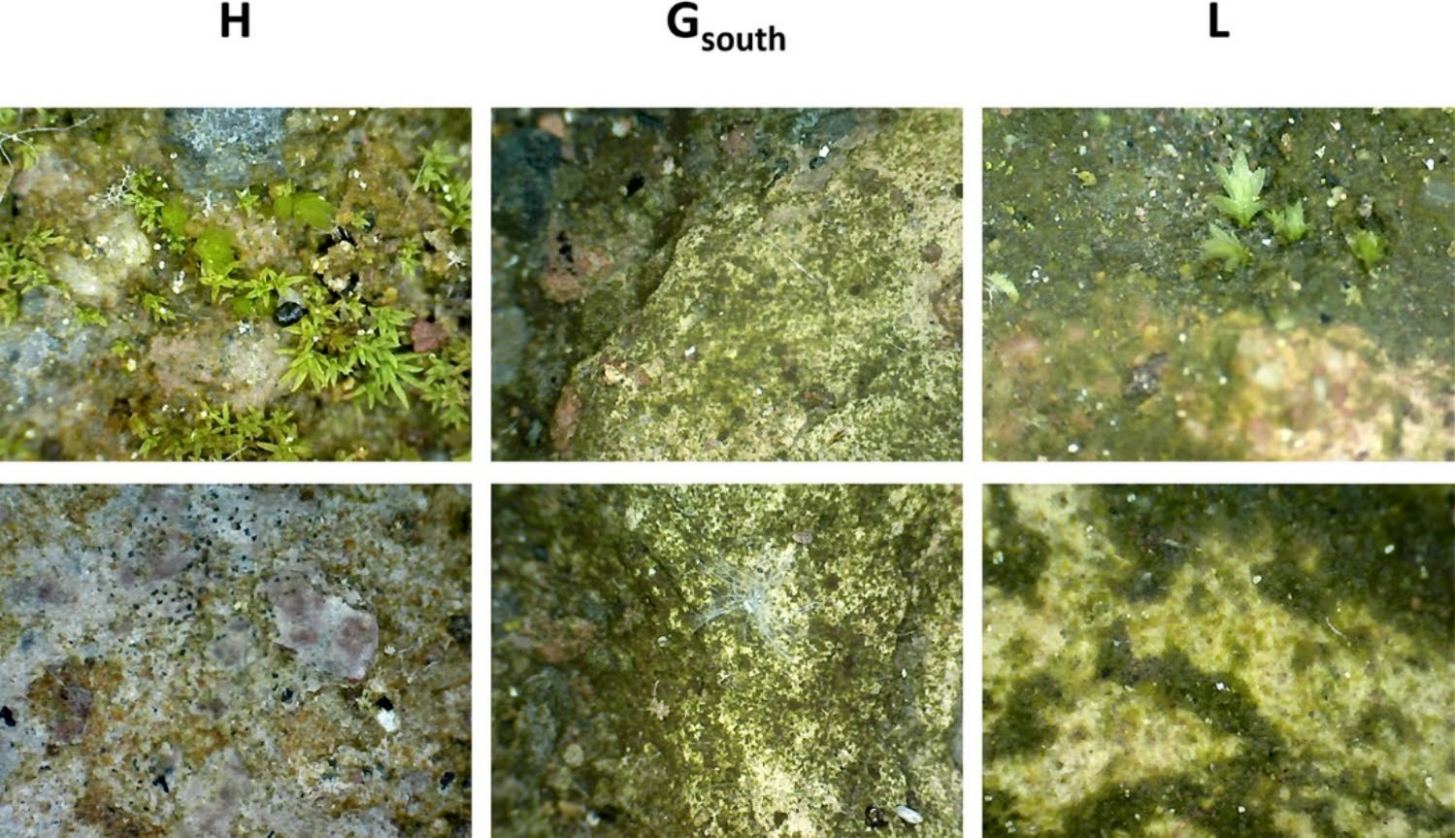
Mic-Fi digital image of biofilm in H, G-south and L sites at different magnifications (5 × 40 and 5 × 100). It can be noted the presence of complex biofilm and bryophytes

Since December 2022, in collaboration with the “Parco archeologico del Colosseo”, the three selected areas have been delimited by the restorers and left untreated, in order to allow the biofilm to grow undisturbed and to compare it with the area subjected to traditional treatments involving a combination of chemical biocides (BIOBAN™ 104 Antimicrobial; Dow Chemical Company, MI, USA) and water washing. Biofilms colonizing the three areas were observed in situ using a digital microscope (Mic-Fi MICFIUVW, Rivoli, Turin, Italy), allowing an initial assessment of biofilm composition and spatial distribution (Fig. 2). In May 2023, biofilm samples were collected from different areas by gently scraping the biomass with a sterile scalpel. Biomass was stored in 15-ml sterile Falcon™ tubes and kept at 4 °C during transport to the laboratory and until processing.

### Microorganisms’ characterization and identification

To characterize the phototrophic fraction of the biofilm, biomass collected from the three sampling sites was mixed using a sterile loop and analyzed by microscopic observation using a Zeiss Axio Scope Light Microscope (LM) at 10 × and 40 × magnification, equipped with a Canon EOS 1300D-W digital camera (Canon S.P.A., Italy). Images were acquired using the EOS Utility software 3.18.40, (Canon S.P.A, Italy). The mixed biomass was suspended in BG11 liquid culture medium (Rippka et al. 1979) to promote the growth and the enrichment in phototrophic microorganisms. Cultures were maintained under controlled conditions (22 ± 2 °C; irradiance of 30-μmol photons m^−2^ s^−1^; 12 h light/dark photoperiod) and monitored by optical density measurements (ONDA-UV spectrophotometer) at 665 nm (OD665). The grown biomass was observed at LM to determine predominant species and analyze for comparison of taxonomic characteristics with the book ‘The Freshwater Algal Flora of the British Isle’ (John et al. 2003) and the website database ‘www.algaebase.org’.

To characterize the heterotrophic cultivable microorganisms, the biomass from the three sites was suspended in sodium pyrophosphate (0.1% w/v) at a final ratio of 1:100, transferred to sterile Erlenmeyer flasks and put in orbital shaker at 150 rpm for 1 h at 28 °C. The suspension was used for colony forming units (CFU) counting by the serial dilution method (Avishai and Davidson 2014; Koch 1883). For each suspension, 100 mL was plated on tryptic soy agar medium (TSA) for heterotrophic bacteria and on potato dextrose (PDA) and maltose extract agar media (MEA) for fungi and incubated at room temperature until colonies appeared in a static incubator (Memmert IPP55, Germany). Bacterial and fungal strains were isolated through the observation of different morphotypes under a stereo microscope Wild Heerbrugg (Germany). The strains were then transferred onto agar medium until pure cultures were obtained. The bacterial strains were identified by the Biolog Lab Services method, using GEN III MicroPlate™ on Biolog Microstation (Biolog Lab Services, Hayward, CA, USA), and molecular identification through the extraction and sequencing of 16S-rDNA by using 9bmf as forward and 1512r as reverse primers (Mühling et al. 2008). Sequence identities were analyzed using the National Center for Biotechnology Information (NCBI) BLAST program (https://blast.ncbi.nlm.nih.gov/Blast.cgi) and the GenBank database. Sequences were deposited in the GenBank database. The fungal isolates were identified by the Biolog Lab Services method, using Biolog FF MicroPlate.

### Gas chromatography–mass spectrometry analysis of EOs composition

Six commercial essential oils extracted by steam distillation from different plants were selected to study their efficacy as alternative green biocides. The EOs used were *Ocimum basilicum* L. (B, basil), *Cinnamomum verum* Presl (C, cinnamon), *Origanum vulgare* L. (O, oregano), *Thymus vulgaris* L. (T, thyme) and *Melaleuca alternifolia* Maiden & Betche (TT, tea tree) oils from L’Aromoteca S.a.S. (Assago, Milan, Italy), and *Lavandula angustifolia* Mill. (L, lavender) oil from Sarandrea Marco E C. S.r.l. (Collepardo, Frosinone, Italy).

The composition of the EOs was assessed by performing chromatographic analyses using a ThermoFischer Trace 1300 series instrument equipped with a Zebron ZB-5MSplus (Phenomenex) (30 m × 0.25 mm, thickness 0.25 μm) fused-silica capillary column and a ThermoFischer ISQ LT mass spectrometer (MS) detector. A 1 μL sample of EO solution (1:200 in n-hexane) was injected into the GC using a split ratio of 50:1, and all the analyses were performed in triplicate. The GC oven was maintained at 60 °C for 5 min, followed by a 4 °C/min ramp up to 200 °C, held for 2 min. The temperature was increased to 280 °C at 35 °C/min and then held for 5 min. Retention indices of the EO components were determined based on the observed retention times of the retention index standard (aliphatic hydrocarbons C4–C24) analyzed under the same temperature program. Chromatography-grade helium was used as the carrier gas at a constant flow of 1 mL/min. Mass spectra of each compound were obtained by electron impact (EI) at 70 eV (scanning from 25 to 700 m/z), and the components of the EOs were identified by comparing their mass profiles and calculated retention indices to those of the NIST (National Institute of Standards and Technology) Library 14 loaded in the detection software of the instrument (Xcalibur software).

### EOs biocidal effect on phototrophs

The EOs were tested at 0.5% and 5% (v/v) concentrations as biocides against phototrophic microorganisms. This range was chosen as previous research has shown that EOs was effective at concentrations between 5 and 1% (v/v) (Bruno et al. 2019; Gabriele et al. 2023; Ranaldi et al. 2022). The range applied was chosen in order to assess the possibility of further reducing the minimum applicable concentration and to compare the results with previous studies. The solutions were prepared in distilled water (DM) and TWEEN20 (Sigma-Aldrich) (1% v/v).

BG11 biomass was centrifuged (10 min at 5000 g) and the supernatant discharged. One millilitre of the biomass (OD = 2.648 ± 0.056) was transferred to BG11 agar Petri dishes and incubated in growth chamber for 2 months (T = 22 ± 2 °C; irradiance = 10 μmol photons m^2^/s⁻^1^; photoperiod: 12 h light/dark), until the surface was fully covered with the phototrophic biofilm. According to Ranaldi et al. (2022), circular fragments (~ 1.5 cm in diameter) were cut from the biofilm and transferred to new agar plates. These plates were maintained under the same controlled conditions throughout the experiment. The EOs were applied by pipetting 200 μL of each one (~ 1 μL/cm^2^) onto the biofilm on the Petri dishes. Two different classes of commercially available biocides were used as positive controls: BIOBAN™ 104 Antimicrobial (CTRL + B) (Dow Chemical Company, MI, USA.), concentrated, stable blend of octylisothiazolinone and quaternary ammonium compound (QUAC-OIT), was tested as 3% (v/v) solution in DM, and ESSENZIO© (CTRL + E) (IBIX BIOCARE, Lugo, Ravenna, Italy), a blend of oregano and thyme EOs, tested as supplied (100% v/v). DM was used as negative control (CTRL −). Tests were performed in triplicate. The efficacy of each treatment was assessed by microscope observation and measuring photosynthetic activity using a portable pulse amplitude fluorometer (PAM).

### Microscopy observation

Light microscopy (LM) was used to evaluate changes in biofilm structure and microorganism integrity (cells colour and morphology) after the treatment with the EOs solutions. The confocal laser scanning microscope (CLSM) FV1000 (Olympus Corp., Tokyo, Japan) with a 60 × oil immersion objective and a numerical aperture (N.A) of 1.35 was used to confirm the viability of samples treated with 5% EOs on agar plates and to measure cell diameters. The phototrophic microorganisms were observed according to their natural pigment autofluorescence by excitation of the chlorophyll with the solid-state laser (excitation: 635 nm; emission 668 nm). The grey channel laser (Ar) with excitation at 488 nm and emission from 480 to 495 nm was used to acquire the image of dead microalgae, in which the autofluorescence was no longer present. 3D images were constructed from a series of 2D cross-sectional images (x–y plane) using IMARIS 6.2.0 software (Bitplane AG Zurich, Switzerland).

### Measurement of photosynthetic activity

A portable pulse amplitude fluorometer (Mini-PAM) (Walz GmbH, Effeltrich, Germany) combined with WinControl software was used to evaluate the effect of EOs on photosynthetic activity. To ensure maximum potential quantum yield, samples were dark adapted for 30 min prior to each measurement. All analyses were performed in a dark room, and the probe was equipped with a holder that held the fibre 6 mm from the biofilm (Rugnini et al. 2020). The yield value was calculated as follows:

$$\mathrm{Yield}=\frac{Fm-Fo}{Fm}$$

where *F*_0_ is the minimum and *F*_*m*_ is the maximum fluorescence of the dark-adapted sample (Bilger et al. 1995; Schreiber and Bilger 1993). The efficacy of EOs at concentrations of 5% and 0.5% against photosynthetic microorganisms growing on agar plates was quantified by determining the maximum quantum yield of photosynthesis on each triplicate sample before each treatment (*t*_0_) and after 24 h (*t*_24h_), 1 week (*t*_1w_), 1 month (*t*_1m_) and 2 months (*t*_2m_).

### Biostatic effect of EOs on heterotrophs

The essential oils found to be effective on phototrophic organisms were tested on the cultivable heterotrophic component of the biofilm. The inhibitory effect of cinnamon (C), oregano (O) and thyme (T) EOs at 5% (v/v) on heterotrophic bacterial and fungal strains were tested according to Antonelli et al. (2020) on Thermo Scientific™ 6 Well Plate (Fisher Scientific Italia, Segrate, Milan, Italy), filled with TSA for the bacteria and MEA for the fungal strains.

Each bacterial strain was suspended in sodium pyrophosphate (0.1% w/v) at a final concentration of 10^8^ CFU/mL and inoculated by spreading 100 mL of bacterial suspension in each well. For fungal strains, spores were collected from agar plates, resuspended in sodium pyrophosphate (0.1% w/v) to reach a concentration of 10^8^ spores/mL and inoculated by spreading the suspension in the well.

To avoid any potential cross-effect resulting from the highly volatile nature of the tested substances, a distinct plate was used for each treatment. As in the photoautotroph tests, 10 mL (~ 1 mL/cm^2^) of each solution to be tested was applied in each well.

According to that done for photoautotrophic biofilm, for heterotrophs, two biocides were used as positive controls: benzalkonium chloride a.s. (QUAC) (CTRL + B) (Antichita Belsito srl, Rome, Italy) was tested as 3% (v/v) solution in DM, and ESSENZIO^©^ (CTRL + E) (IBIX BIOCARE, Lugo, Ravenna, Italy), a blend of oregano and thyme EOs, tested as supplied (100% v/v). DM was used as negative control (CTRL −). The plates were incubated at 28 °C for 7–10 days. The inhibitory effect was evaluated by monitoring the growth of microorganisms into the treated wells by observations under a stereo microscope and assigning a positive ( +), negative ( −) or partial (+ / −) score based on the presence, absence and intermediate growth, respectively. If the microorganisms occupied the whole area of well, + , if just a portion of well, + / − and if there was no growth at all, − .

### Statistical analysis

All measurements with PAM were conducted in triplicates, and the results were reported as the mean ± SD (standard deviation) values. The statistical analyses were performed using GraphPad Prism 10.2.3. Parametric tests (ordinary one-way ANOVA) were applied to investigate the data and compare them to one another. Moreover, Tukey’s multiple comparisons test was also applied. Values of *p* < 0.05 were considered statistically significant. The same test was applied to investigate the differences in cell diameter after the treatment with EOs (*N* ≥ 26), with data acquired from CLSM.

## Results

### Biofilm characterization

**Fig. 3:**
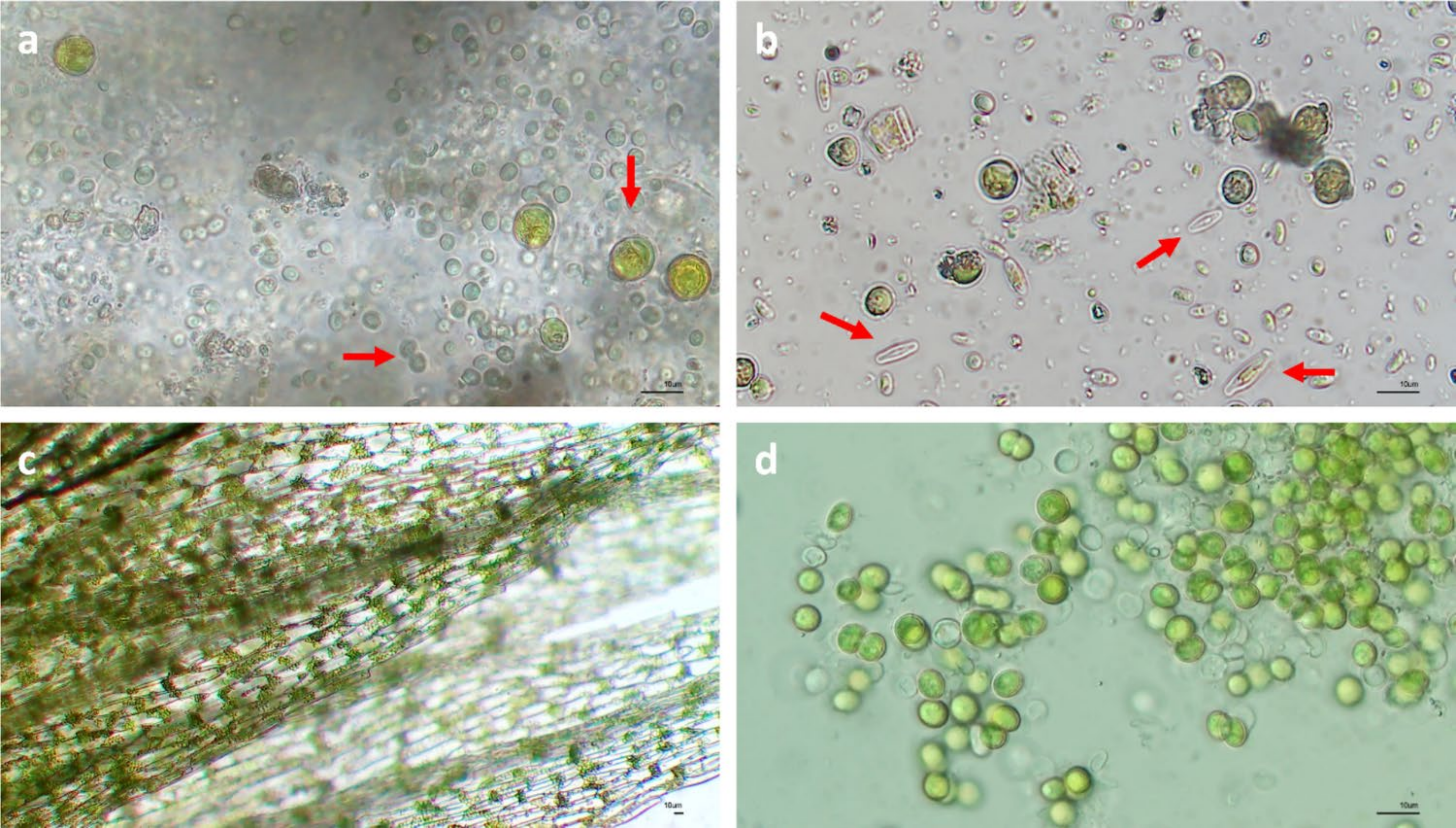
Images of microorganisms forming the biofilm sampled in the Colosseum hypogeum: **a** cyanobacteria and green microalgae; **b** green microalgae and diatoms; **c** mosses. **d** The predominant phototrophic microorganism belonging to the order of Chlorellales grown in BG11 medium under controlled culture conditions. The red arrows indicate the different species of microorganisms. The photos were acquired at LM with 40 × (a, b and d) and 10 × (c) objectives. Bar = 10 µm

The LM observation of the biofilm sampled in the hypogeum of the Colosseum showed that the phototrophic community was composed of cyanobacteria (Fig. 3a), green microalgae (Fig. 3a, b), diatoms (Fig. 3b) and mosses (Fig. 3c). Specifically, site H was predominantly colonized by mosses, attributed to its direct exposure to sunlight throughout the day, which significantly promotes their growth. Nevertheless, various species of green microalgae and diatoms were also observed in this site. Conversely, site G-south was heavily influenced by the proliferation of microalgae, whereas site L exhibited a more complex community structure, including green microalgae, diatoms and bryophytes (Fig. 2). Nevertheless, after enrichment of the cultures in BG11 medium, the biofilm was mainly formed by green microalgae belonging to the phylum Chlorophyta, the class Trebouxiophyceae and the order Chlorellales (Fig. 3d). The presence of chlorophyll *a* and *b* gives the coccoid cells their typical bright green colour. However, under high irradiation they can appear orange due to the production of high levels of carotenoids. They are spherical in shape and have a diameter ranging from 4.4 to 7.1 µm. The cell has a thin wall and contains a large chloroplast.

The heterotrophic microbial load ranged from 1.6 × 10^4^ in the H site to 2.5 × 10^4^ CFU/g biomass in G-south and L sites. A total of 22 bacterial and six fungal strains were isolated from the culturable heterotrophic microbial fraction (Table 1). The bacteria are predominantly gram-negative and belong to the a- and g-Proteobacteria and Flavobacteria classes. The only gram-positive Bacillus, *Exiguobacterium mexicanum*, was isolated from the G-south site. The only species detected at all sampling points was *Enterobacter quasihomaechei*. The isolated fungi belong to the genera *Fusarium*, *Penicillium* and *Paecilomyces.*

**Table 1:** Bacterial and fungal strains isolated from the three sampling sites by plating on TSA, PDA and MEA media. Gene bank accession number for bacterial 16S sequences. *Validation of sequences in progress

Strain		H	G-south	L	Gene Bank accession number
Bacteria					
COL 1.3	*Acinetobacter junii*	x			PQ416651
COL 3.2	*Aeromonas rivipollensis*			x	PQ416660
COL 4.8	*Chryseobacterium contaminans*			x	not deposited*
COL 2.4	*Ensifer adhaerens*		x		PQ416656
COL 1.2	*Enterobacter quasihomaechei*	x	x	x	PQ416650
COL 1.1	*Enterobacter sichuanensis*	x			PQ416649
COL 3.8	*Enterobacter sp.*			x	not deposited*
COL 2.2	*Exiguobacterium mexicanum*		x		PQ416654
COL 4.6	*Pantoea agglomerans*			x	PQ416665
COL 2.7	*Pantoea ananatis*		x		PQ416658
COL 2.3	*Pseudomonas alkilphenolica*		x		PQ416655
COL 1.4	*Pseudomonas chengduensis*	x			PQ416652
COL 3.4	*Pseudomonas lalkuanensis*			x	PQ416661
COL 2.1	*Pseudomonas mosselii*		x		PQ416653
COL 3.6	*Pseudomonas oryzihabitans*			x	PQ416662
COL 3.3	*Pseudomonas resinovorans*			x	Not deposited*
COL 2.5	*Pseudomonas sediminis*		x	x	PQ416657
COL 4.7	*Pseudomonas soli*			x	PQ416666
COL 4.3	*Serratia liquefaciens*			x	PQ416664
COL 2.8	*Serratia rubidaea*		x		PQ416659
COL 4.1	*Shigella flexneri*			x	PQ416663
COL 4.10	*Stenotrophomonas lactitubi*			x	PQ416667
Fungi					
COL 4.16	*Fusarium chlamydosporium*			x	
COL 2.12	*Paecilomyces lilacinus*		x	x	
COL 2.11	*Penicillium chrysogenum*		x		
COL 4.13	*Penicillium citreonigrum*			x	
COL 4.14	*Penicillium miczynskii*			x	

### GC–MS analysis

The compositional profile of *Lavandula angustifolia* Mill. (the same batch used for this work) was reported in our previous study, in which 28 molecules were identified, among which the main compound are terpenoids characterized by the presence of an oxygenated function (Ranaldi et al. 2022). Linalool is the linear terpenoid that primarily constitutes the lavender EO with a relative abundance of 42.2%. Conversely, the other main constituents, such as camphor (13.0%), eucalyptol (11.5%), borneol (7.4%) and ocimene (5.7%), are all characterized by a cyclic structure. The results of the GC/MS analysis of the other five essential oils are reported in Table 2, which lists all components with a relative abundance greater than 0.1%.

**Table 2:** Chemical characterization of *O. basilicum* (B), *C. verum* (C), *O. vulgare* (O), *T. vulgaris* (T), and *M. alternifolia* (TT) EOs. Calculated retention indices and relative abundance percentages (> 0.1%) of the compounds detected by GC/MS analysis

Compound	RI	Area %				
		B	C	O	T	TT
α-Thujene	926					0.7
α-Pinene	931		3.2		1.3	2.0
Camphene	948		1.1			
Benzaldehyde	959		0.2			
α-Sabinene	973					0.1
β-Pinene	975	0.1	1.2	0.2	0.7	0.6
1-Octen-3-ol	977			0.1		
β-Myrcene	987			0.1	1.6	0.6
α-Phellandrene	1007					0.2
α-Terpinene	1015			0.3	0.3	9.3
*p*-Cymene	1023		0.2	7.3	27.3	3.9
Limonene	1027			0.4		1.0
Eucalyptol (1,8-cineole)	1031	3.1	2.8	1.1		4.6
trans-b-ocymene	1046	0.4				
γ-Terpinene	1056			3.3	6.8	19.8
Isoterpinolene	1085					3.3
Linalool	1098	1.1		3.0	4.5	
cis-2-Menthenol	1125					0.2
cis-β-Terpineol	1143					0.1
Camphor	1145			0.7		
Borneol	1170			0.8		
Terpinen-4-ol	1178	0.2	0.5	0.5	0.3	48.5
α-Terpineol	1192		0.6	0.6		3.6
Estragole	1198	92.1				
(Z)-Cinnamaldehyde	1215		0.4			
Fenchyl acetate	1219	0.2				
(E)-Cinnamaldehyde	1272		72.4			
Bornyl acetate	1282		0.7			
Isobornyl acetate	1284		0.8			
Thymol	1288			2.4	54.5	
Carvacrol	1297			76.1	2.0	
Eugenol	1348		5.1			
α-Copaene	1373		1.8			
β-Elemene	1386	0.2				
Methyleugenol	1396	1.0				
β-Caryophyllene	1416		1.9	2.3	0.7	
α-Bergamotene	1430	0.8				
Aromandendrene	1438					0.4
(E)-Cinnamyl acetate	1442		6.9			
α-Caryophyllene	1452			0.1		
Alloaromadendrene	1459					0.1
Ledene (Viridiflorene)	1490					0.3
β-Cyclogermacrane	1495					0.1
γ-Cadinene	1509	0.2				
Eugenyl acetate	1510		0.3			
β-Cadinene	1517					0.4
Caryophyllene oxide	1578			0.6	0.2	
Globulol	1585					0.1
τ-Cadinol	1638	0.5				

As shown in Table 2, all the studied EOs are complex mixtures of several compounds, ranging from 12 to 22 molecules each. Basil (B) and cinnamon (C) EOs are the only two extracts mainly constituted by non-terpene or non-terpenoid derivatives. In fact, basil EO is almost exclusively composed of estragole, while cinnamaldehyde, cinnamyl acetate, and eugenol are the main constituents of cinnamon EO.

Oregano (O) and thyme (T) EOs have a very similar pattern of constituents, sharing 11 of their components. Another similarity between these two extracts is the presence of *p*-cimene as their second most abundant principle. It is a cyclic diterpene and represents the aromatic skeleton of the main constituent of both oregano and thyme EOs: carvacrol and thymol, respectively.

Despite the tea tree (TT) EO is also composed primarily of cyclic terpenes and terpenoids, all of its most abundant compounds are non-aromatics. In fact, both α-terpinene and γ-terpinene, the secondary principles constituting tea tree EO, are reduced forms of *p*-cimene. In addition, terpinen-4-ol, its most abundant compound, could derive from the hydration of the two terpinene-like products mentioned above.

### Biocidal effect of EOs on phototrophs

**Fig. 4:**
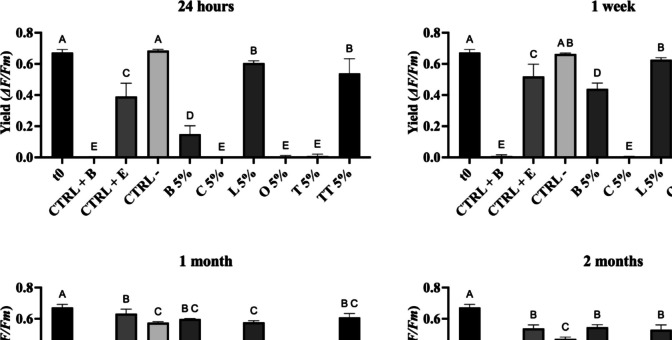
Photosynthetic yields at different time from the application of *O. basilicum* (**B**), *C. verum* (**C**), *L. angustifolia* (L), *O. vulgare* (O), *T. vulgaris* (T) and *M. alternifolia* (TT) EOs at 5% against phototrophic biofilms. The positive controls were treated with BIOBAN 104 (CTRL + B) and ESSENZIO (CTRL + E), while the negative control was treated with DM (CTRL −). Results are represented by their mean ± SD values. Different letters in each column indicate significant differences (*p* < 0.05)

Experiments were carried out on phototrophic biofilm grown on BG11 agar to compare the biocidal activity of the six EOs at concentrations of 5% and 0.5% (v/v). Photosynthetic activity was measured over time after the treatments. EOs at a concentration of 0.5% (v/v) were not effective as 5% concentration under the tested conditions, because EOs at 0.5% did not significantly impact the biofilm; the results are reported in Online Resource (Table S1). Tests with 5% (v/v) EOs have proven their effectiveness (Fig. 4).

Twenty-four hours after the application of the green biocides, cinnamon (C), oregano (O) and thyme (T) EOs reduced the photosynthetic yield by up to 99%. This result was comparable with those obtained with the BIOBAN™ 104 (CTRL + B), in which the yield value dropped from 0.673 ± 0.019 to 0.002 ± 0.003. Basil EO (B) reduced yield by 76% compared to time zero, a result statistically different from all treatments. Lavender (L) and tea tree (TT) EOs only slightly reduced photosynthetic activity. ESSENZIO (CTRL + E), the second positive control, was not very effective, resulting in a minimal reduction compared to time zero (0.390 ± 0.086), which was statistically different from that of the DM-treated negative control.

After 1 week, the photosynthetic yield of the samples treated with cinnamon (C), oregano (O) and thyme (T) remained close to zero, as did that of BIOBAN (CTRL + B) (Fig. 4), whereas a partial recovery of biofilm viability was observed in the basil EO-treated samples, similar to that of the samples treated with ESSENZIO (CTRL + E). Lavender (L) and tea tree (TT) EO showed no statistical differences compared to the negative control (CTRL −).

After one and two months, no recovery in photosynthetic activity was observed only in samples treated with cinnamon (C), oregano (O), thyme (T) EOs and BIOBAN (CTRL + B) (*p* > 0.05; Fig. 4).

### Biofilm observations

**Fig. 5:**
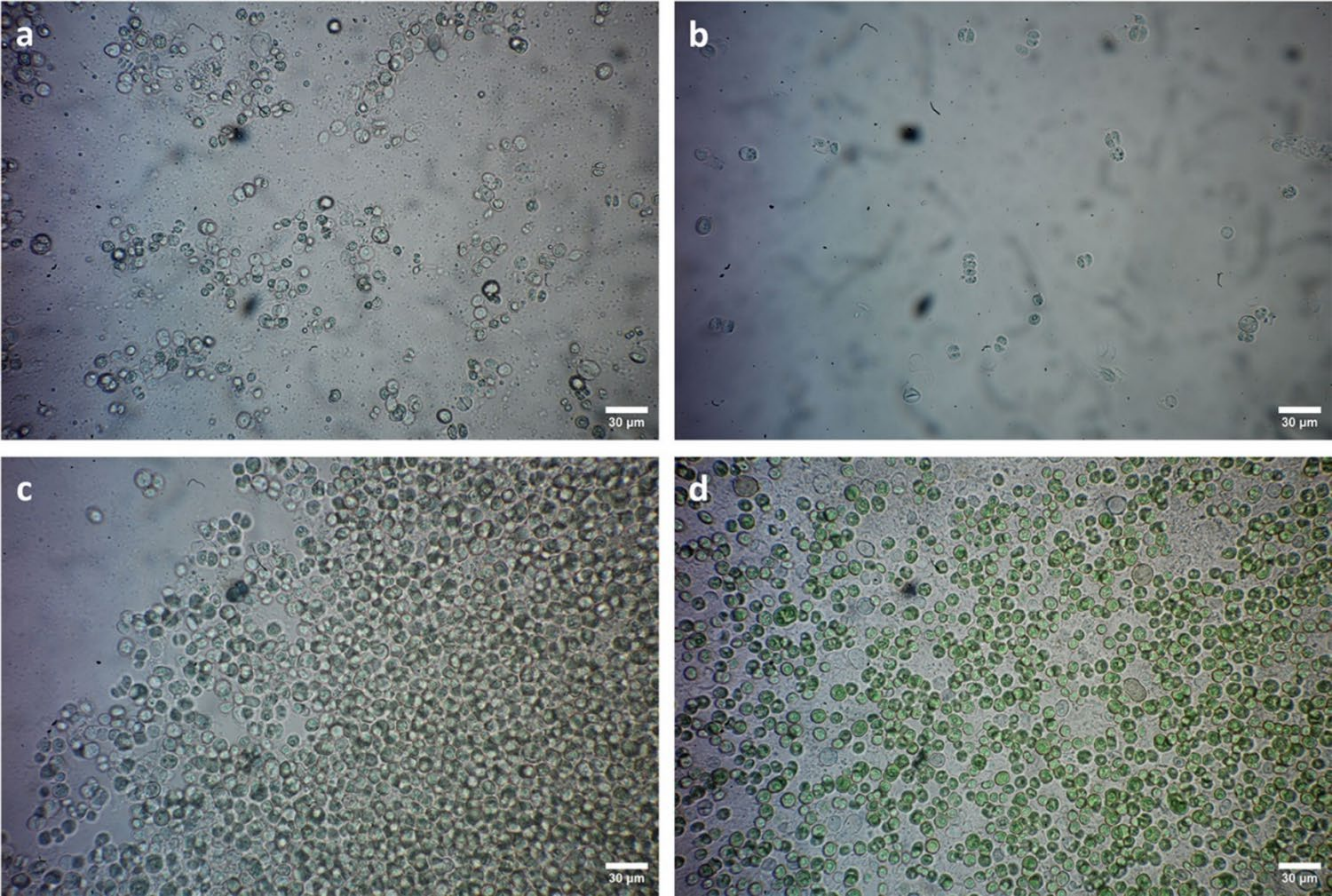
LM images of biofilms grown on agar plates two months after treatment with 5% EOs of **a** *C. verum*, **b** *O. vulgare*, **c** *T. vulgaris*, **d** negative control (DM). Bar = 30 µm

Two months after the application of 5% (v/v) EOs, the biofilm was observed under light microscopy (LM) in order to assess whether there were any observable changes in cell morphology (Fig. 5). Cinnamon and oregano EO-treated biofilms appeared colourless and devoid of photosynthetic pigments (Fig. 5a–b), indicating a loss of pigments due to biocide application, which confirms the findings of PAM measurements. Similarly, microorganisms treated with thyme EO showed a lighter, paler appearance (Fig. 5c). In contrast, in the negative control (Fig. 5d), cells displayed a bright green colour, confirming the vitality of the phototrophic microorganisms.

**Fig. 6:**
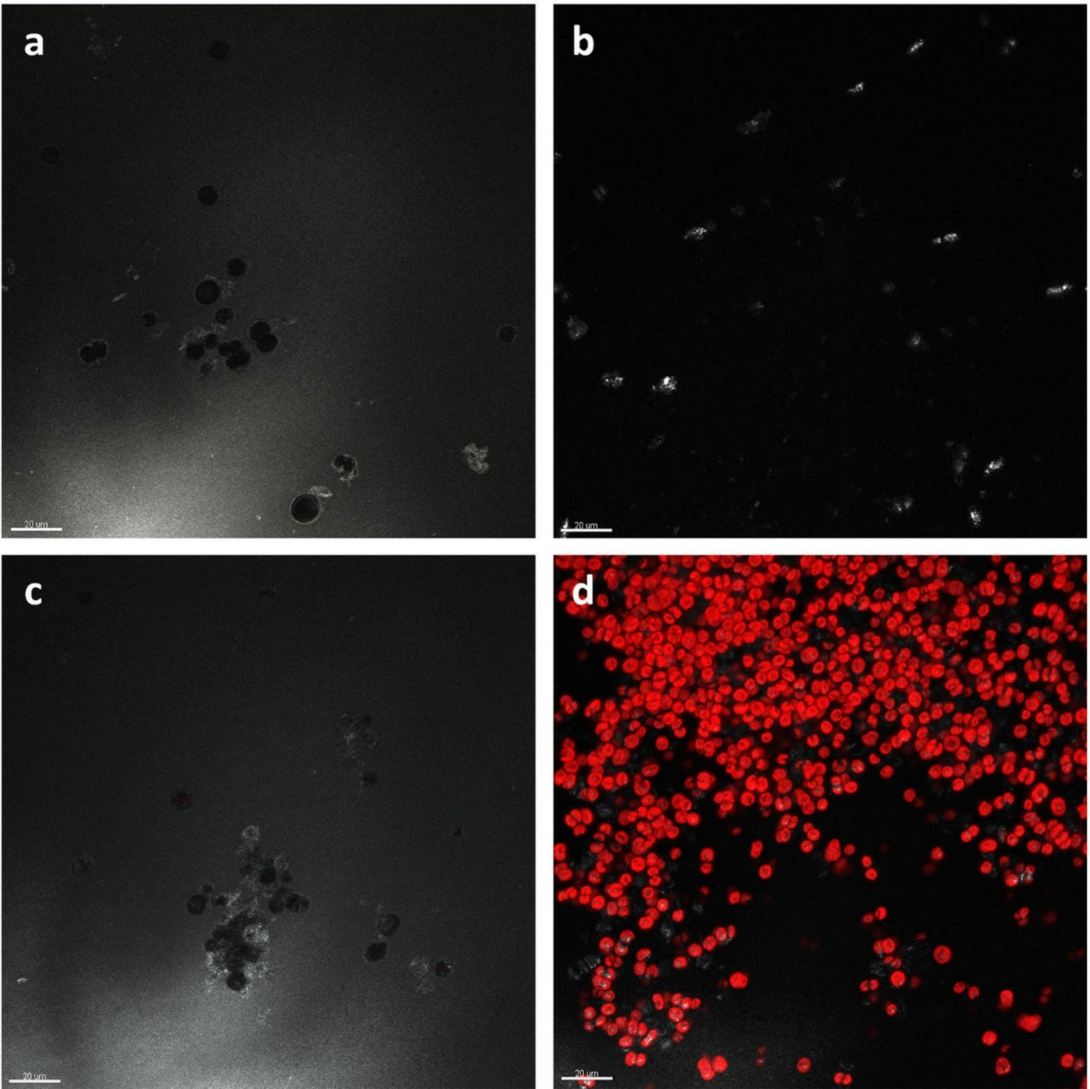
CLSM images of biofilm treated with **a** *C. verum*, **b** *O. vulgare* and **c** *T. vulgaris* EOs at 5% showing the absence of autofluorescence in biofilm forming cells compared to **d** negative control. Bar = 20 µm

Pigment loss was also observed by CLSM, using the grey channel laser (488 nm) to capture images of dead microalgae (Fig. 6). Biofilms treated with cinnamon, oregano and thyme EOs had no living cells (Fig. 6a–c), due to the fact that the chlorophyll *a* autofluorescence was completely absent. In the negative control (Fig. 6d), cells exhibited natural phototrophic autofluorescence with an excitation at 635 nm, corresponding to the presence of chlorophyll *a*, indicating the vitality of the biofilm. Additionally, cell diameters were measured to assess morphological changes induced by the 5% biocide application. Cells treated with distilled water (CTRL −) had a diameter of 5.817 ± 0.565 µm (*N* = 30), which was statistically similar to those treated with oregano and BIOBAN (*p* > 0.05). In contrast, cells treated with cinnamon and thyme EOs showed significantly larger diameters, with values of 7.316 ± 1.223 µm (*N* = 26) and 6.865 ± 1.151 µm (*N* = 30), respectively, exhibiting statistically significant differences compared to both the negative and positive controls (*p* < 0.0001).

### Biostatic effect of EOs against heterotrophic microorganism

The results of the inhibitory effect of cinnamon, oregano and thyme EOs at 5% (v/v) on heterotrophic bacterial and fungal are reported in Table 3. Concerning bacteria, cinnamon EO was the most effective followed by oregano and thyme EOs*. Acinetobacter junii*, *Enterobacter sichuanensis* and *Exiguobactrium mexicanum* were sensitive both to EOs and benzalkonium chloride. *Aeromonas rivipollensis* was resistant to all the EOs tested but not to the benzalkonium chloride. Conversely, *Chryseobacterium contaminans* and *Ensifer adhaerens* were sensitive to all EOs but were resistant to benzalkonium chloride (CTRL + B) and ESSENZIO (CTRL + E). *Pseudomonas alkilphenolica*, *Pseudomonas mosselii*, *Pseudomonas sediminis*, and *Pseudomonas soli* were resistant to all the treatments tested, even to the positive controls. Numerous strains belonging to the phylum γ-proteobacteria (*Pantoea*, *Pseudomonas*, *Serratia*, *Shighella*) showed resistance to benzalkonium chloride (CTRL + B) but exhibited sensitivity to one or more essential oils. The tested EOs were effective against all the fungal strains isolated that were resistant to the benzalkonium chloride (CTRL + B) at the tested concentration.

**Table 3:** The inhibitory effect of tested EOs and controls against microbial strains isolated from Colosseum hypogeum. Positive ( +), negative ( −) or intermediate (+ / −) reflect the presence, absence and partial growth, respectively. Cinnamon (C), oregano (O) and thyme (T)

Strain		CTRL + B	CTRL + E	CTRL –	C	O	T
Bacteria							
COL 1.3	*Acinetobacter junii*	−	+	+	−	−	−
COL 3.2	*Aeromonas rivipollensis*	−	+	+	+	+	+
COL 4.8	*Chryseobacterium contaminans*	+ / −	+	+	−	−	−
COL 2.4	*Ensifer adhaerens*	+	+	+	−	−	−
COL 1.2	*Enterobacter quasihomaechei*	−	+	+	−	−	+
COL 1.1	*Enterobacter sichuanensis*	−	+	+	−	−	−
COL 3.8	*Enterobacter sp.*	−	+	+	−	−	+
COL 2.2	*Exiguobacterium mexicanum*	−	+	+	−	−	−
COL 4.6	*Pantoea agglomerans*	+	+	+	−	+	+
COL 2.7	*Pantoea ananatis*	+ / −	+	+	−	+ / −	+
COL 2.3	*Pseudomonas alkilphenolica*	+ / −	+	+	+	+	+
COL 1.4	*Pseudomonas chengduensis*	+ / −	+	+	−	+ / −	+
COL 3.4	*Pseudomonas lalkuanensis*	+	+	+	−	+	+
COL 2.1	*Pseudomonas mosselii*	+	+	+	+	+	+
COL 3.6	*Pseudomonas oryzihabitans*	+	+	+	−	−	+
COL 3.3	*Pseudomonas resinovorans*	+	+	+	−	+	−
COL 2.5	*Pseudomonas sediminis*	+	+	+	+	+	+
COL 4.7	*Pseudomonas soli*	+	+	+	+	+	+
COL 4.3	*Serratia liquefaciens*	+ / −	+	+	+	−	+ / −
COL 2.8	*Serratia rubidaea*	+ / −	+	+	−	−	+
COL 4.1	*Shigella flexneri*	+	+	+	+	−	−
Fungi							
COL 4.16	*Fusarium chlamydosporium*	+	+	+	−	−	−
COL 2.12	*Paecilomyces lilacinus*	+	+	+	−	−	−
COL 2.11	*Penicillium chrysogenum*	+	+	+	−	−	−
COL 4.13	*Penicillium citreonigrum*	+	+	+	−	−	−
COL 4.14	*Penicillium miczynskii*	+	+	+	−	−	−

## Discussion

The growth of biodeteriogens is a natural ecological phenomenon, driven by favourable environmental factors, such as irradiation, temperature and relative humidity, which fluctuate throughout the day and across the seasons (Liu et al. 2018; Yu et al. 2024). Over the years, repeated treatments with traditional chemical biocides have led to the development of microbial resistance. Therefore, to counteract the growth of biodeteriogenic biofilm, it is essential to gradually increase the concentration of these biocides and shorten the intervals between treatments. Furthermore, chemical treatments are combined with physical cleaning with a high-pressure spray water that does not remove necrotic biomass and spreads microorganisms that spontaneously regenerate in an even shorter time frame, especially if environmental conditions are favourable. This research is focused at identifying one or more broad-spectrum EOs that could be a valuable tool in addressing complex microbial biofilms with the aim of finding biocides that, thanks to their complex composition, could avoid the selection of resistant species. For this reason, microorganisms forming the biofilms sampled in the hypogeum of the Colosseum were collected and used as target species in lab experiments to evaluate the potential use of six different EOs as eco-friendly alternatives biocides to replace traditional chemicals. EOs are known to be mixtures of different organic compounds. Their composition depends on many factors, such as the genotype (species and cultivation), the geographical origin, the soil characteristics, the climatic conditions and the season of collection, the extraction method and the plant parts (flower, seed, fruit, etc.) used to extract the essential oil. This means that the same EO may act differently on the same microorganism depending on its composition (Bakkali et al. 2008; Cappitelli et al. 2020). The main components of the six EOs tested were very similar to those reported in the literature (Caprari et al. 2021; Liyanage et al. 2017; Raina and Kumar 2017; Santos et al. 2014; Zinno et al. 2023), but several chemotypes of all the selected EOs were identified and their composition is quite different.

Here, a preliminary study of the effect of the selected EOs was initially performed on phototrophic microorganisms with the aim of selecting the most effective ones and evaluating their efficacy also against the heterotrophic component of the biofilm. Each oil was diluted in 1% Tween 20 in water to obtain a homogeneous solution without affecting the vitality of the microorganisms, as reported by Ranaldi et al. (2022) for cyanobacterial biofilms.

Tea tree, lavender and basil EOs at 5% (v/v) resulted almost ineffective against the microalgal biofilm. This result is in accordance with literature data, which report that these essential oils show significant efficacy only when used in combination with other biocidal approaches. In fact, *Lavandula angustifolia* EO (5%) did not affect the viability of biofilms composed of a mixture of filamentous biodeteriogenic cyanobacteria in hypogean environments (Ranaldi et al. 2022), but its biocidal efficiency was enhanced when combined with other plant extract, such as *Thymus vulgaris* EO or alcoholic leaf extracts of liquorice (*Glycyrrhiza glabra*) (Bruno et al. 2019; Rugnini et al. 2020). Similarly, basil EO reduced photosynthetic yield immediately after treatment, but the positive effect persisted for a short time (less than one month). Good results were obtained by Macchia et al. (2022) on mosaics in the Archaeological Park of Ostia Antica (Rome) by encapsulating basil and other EOs in agar gel. Conversely, cinnamon, oregano and thyme EOs at 5% concentration induced drastic decrease in the photosynthetic activity of the algal biofilm and their effect was maintained up to two months after the treatments, showing the same efficiency as the BIOBAN positive control.

In addition, cinnamon and thyme were found to be moderately effective against phototrophs also at 0.5% concentration. This preliminary result could be enhanced by encapsulating them into inert matrices to further decrease the concentration of EOs necessary to inhibit the growth of these microorganisms. In fact, as demonstrated in our previous works, thyme EO conveyed by alginate hydrogel was effective even at the 0.1% against cyanobacteria (Gabriele et al. 2023) and at 0.25% towards a complex biopatina growing on the Fortunato Depero’s mosaic of “*Le Professioni e le Arti*”, Rome (Italy) (Bruno et al. 2023). The efficacy of *Cinnamomum cassia* Presl. EO was also demonstrated by Long et al. (2024), who applied an extract of this EO against algal biofilm as well as the *Thymus capitatus* (L.) Hoffmanns & Link that has been successfully applied to counteract cyanobacteria and green algae colonizing outdoor stone surfaces (Candela et al. 2019). Both studies relied primarily on macroscopic observations to assess the vitality of the biofilms and/or colorimetric measure to evaluate any alteration in the substrate, without specifically evaluating the photosynthetic activity. ESSENZIO, a mixture of oregano and thyme EOs and BIOBAN, a quaternary ammonium salt–based biocide, both used by restorers to counteract biological patinas, were selected as positive controls for phototrophic biofilm. ESSENZIO resulted to be completely ineffective against the microalgal biopatina, whereas it had a cleaning action when applied at 50% for the removal of artificial biofilm cultivated on painted samples (Cennamo et al. 2023). Otherwise, good biocidal activity was observed when the target phototrophic organisms were treated with the benzalkonium chloride, a result that was confirmed even two months after treatment. Benzalkonium chloride (as active ingredient and formulation) is often ineffective on many heterotrophic bacteria and target fungi that are known to cause deterioration in cultural heritage and are commonly found in subterranean sites (Gadd et al. 2024; Isola et al. 2021; Sterflinger and Piñar 2013).

Therefore, in this study the biostatic activity of the three EOs, which are efficient biocides for the phototrophic microorganisms, and the two positive controls (ESSENZIO and benzalkonium chloride), was tested on all bacterial and fungal strains isolated in the hypogeum of the Colosseum. The benzalkonium chloride, which was able to remove algae, was ineffective against fungi and most of gram-negative target strains, showing resistance but exhibiting sensitivity to one or more essential oils.

Although Gram-negative bacteria are less susceptible to antibiotics due to their complex cell membranes that limit the penetration of active ingredients, essential oils have demonstrated antibacterial and antifungal properties (Huang et al. 2014). In this study, all the selected EOs resulted effective on most of the strains tested, including strains resistant to benzalkonium chloride, according to that reported in several studies (Ioan et al. 2023; Reale et al. 2024; Russo and Palla 2023; Santo et al. 2023). In particular, cinnamon EO appeared to be the most effective among the EOs investigated here, by inhibiting almost all the heterotrophs isolated except for only five bacterial strains. This evidence highlights its potential use as widespread bioactive substance to replace quaternary ammonium compound (QUAC) in controlling the growth of the microbial biopatina also in situ. The microbial strains in the hypogeum of Colosseum are drug-resistant probably due to repeated treatments with a single active ingredient, intensified in dose and timing and carried out without a detailed characterization of the to be removed. Moreover, it is notable that some of the strains identified as resistant to benzalkonium chloride belong to facultative pathogens (e.g. *Acinetobacter junii*, *Enterobacter* spp., *Pantoea* spp., *Pseudomonas* spp., *Serratia liquefaciens* and *Shigella flexneri*). This highlights the importance of environmental sanitation not only for the preservation of cultural heritage but also from a human health perspective.

Furthermore, benzalkonium chloride has a long carbon chain that limits the penetration of the molecule into the hydrophilic matrix of biofilms, reducing its bactericidal efficiency, e.g. against *P. aeruginosa* biofilms (Campanac et al. 2002). *Cinnamomum* spp., *Thymus* spp*.* and *Origanum* spp*.* EOs have been shown to have antibacterial action against several microorganisms and etiological agents, including *Pseudomonas* spp. (Kavanaugh and Ribbeck 2012). The primary compounds in cinnamon EO, cinnamaldehyde and eugenol, react with lipid and hydroxyl radicals to transform them into stable products (Jayaprakasha et al. 2007), inactivate bacterial cell wall enzymes and block essential enzymatic activities (Di Pasqua et al. 2007). Carvacrol, the main ingredient of oregano EO, alters cell membrane permeability, causing cell death (Fang et al. 2019). Thymol induces the breakdown of bacterial cell membranes (Guimarães et al. 2019). The EOs were effective against all the isolated fungal strains that were resistant to the benzalkonium chloride at the concentration tested. Cinnamon, oregano and thyme EOs are known to be effective against fungi genera affecting cultural heritage such as *Aspergillus* spp., *Penicillium* spp., *Alternaria* spp. and *Cladosporium* spp. (Fidanza and Caneva 2019). The effectiveness of thymol in preserving cultural heritage and its fungicidal properties have been proven since 1975, when the practical and inexpensive thymol chamber was created for fumigating paper and parchment (Byers 1983). This was the first of other methods of using thymol.

Conservation and restoration of stone artefacts must prioritize strategies that avoid damaging to the material (Fernandes 2006). The present work deepens our knowledge of the efficacy of cinnamon, oregano and thyme EOs as biocides against deteriogenic microorganisms, having demonstrated their efficacy against phototrophs and heterotrophs isolated from the hypogeum of the Colosseum. Based on this concept, the next step will be to investigate the interactions between the specific stone substrate and the selected EOs, with the aim of developing a potential eco-friendly biocide suitable for in situ application. In accordance with Otero’s perspective (Otero 2022) and the UN 2030 Agenda for Sustainable Development (UN 2015), heritage conservation is oriented towards more sustainable strategies among which EOs are promoted as green conservation practices for cultural heritage preservation and protection. Moreover, given the diverse susceptibility of different target microorganisms at EOs, the formulation of a mixture of them could be a broad-spectrum EO–based biocide that will be effective against a complex microbial community and also will help to reduce the risk of resistance induction. Furthermore, standardizing the application method, such as embedding in a carrier matrix and definition of the exposure time, would allow the establishment of a reproducible protocol that could provide a sustainable alternative to traditional conservation methods and could be applied directly in situ.

## Supplementary Information

Below is the link to the electronic supplementary material.

Supplementary file1 (PDF 233 KB)

## Data Availability

All data supporting the findings of this study are available within the paper and its Supplementary Information.
