# Supplementary material for: The role of essential oils as eco-friendly strategy to control biofilm collected in the Colosseum (Rome, Italy)
Source: Appl Microbiol Biotechnol. 2025 Feb 18;109:48. doi: 10.1007/s00253-025-13433-1 (PMC11836000; doi:10.1007/s00253-025-13433-1)
Supplement: Supplementary file 1 [file 253_2025_13433_MOESM1_ESM.pdf]

The role of essential oils as eco-friendly strategy to control biofilm collected in the Colosseum (Rome, Italy)

Roberta Ranaldi[1a†*], Lorenza Rugnini[1†], Giada Migliore[2], Flavia Tasso[2], Francesco Gabriele[3], Nicoletta Spreti[3], Francesco Scuderi[1], Roberto Braglia[1], Patrick Di Martino[4], Angelica Pujia[5], Antonella Canini[1].

[1]Department of Biology, Tor Vergata University of Rome, Via della Ricerca Scientifica 1, 00133 Rome, Italy.
[a]PhD program in Evolutionary Biology and Ecology, Department of Biology, Tor Vergata University of Rome, Rome, Italy.
[2]ENEA, Department of Territorial and Production Systems Sustainability, Via Anguillarese 301, 00123 Rome, Italy.
[3]Department of Physical and Chemical Sciences, University of Aquila, Via Vetoio, Coppito, I-67100, L'Aquila, Italy.
[4]ERRMECe Laboratory, University of Cergy-Paris, rue 13 Descartes site de Neuville-sur-Oise, 95031 Cergy-Pontoise, France.
[5]Chief conservator, Parco archeologico del Colosseo, Piazza Santa Maria Nova 53, 00186 Roma
[†] These authors contributed equally to this work.
[*] Corresponding author: ranaldi.roberta@gmail.com

Table S1. Photosynthetic yields of treated biofilms with EOs in the study period, with initial yield value of 0.673 ± 0.019 (t_0). Different superscript letters in each column indicate significant differences ($p < 0.05$) in relation to the different biocides at the same time period. Data are reported as mean ± SD values.

	Yield ($\Delta F/F_m$)			
**Treatment**	t_{24h}	t_{1w}	t_{1m}	t_{2m}
**CTRL + B**	0.002 ± 0.003 ^{A}	0.009 ± 0.008 ^{A}	0.002 ± 0.004 ^{A}	0.000 ± 0.000 ^{A}
**CTRL + E**	0.390 ± 0.086 ^{C}	0.519 ± 0.079 $^{B, C}$	0.633 ± 0.029 $^{B, C}$	0.538 ± 0.023 $^{B, C}$
**CTRL -**	0.685 ± 0.008 ^{E}	0.663 ± 0.006 ^{E}	0.575 ± 0.006 ^{E}	0.471 ± 0.012 $^{B, C}$
**B 5%**	0.149 ± 0.054 ^{B}	0.440 ± 0.037 ^{B}	0.599 ± 0.002 ^{B}	0.546 ± 0.016 ^{C}
**B 0.5%**	0.662 ± 0.015 $^{D, E}$	0.647 ± 0.021 ^{E}	0.600 ± 0.018 ^{E}	0.526 ± 0.014 $^{B, C}$
**C 5%**	0.001 ± 0.002 ^{A}	0.003 ± 0.004 ^{A}	0.001 ± 0.002 ^{A}	0.000 ± 0.000 ^{A}
**C 0.5%**	0.010 ± 0.008 ^{A}	0.035 ± 0.045 ^{A}	0.182 ± 0.263 ^{A}	0.201 ± 0.290 $^{A, C}$
**L 5%**	0.605 ± 0.015 $^{D, E}$	0.627 ± 0.013 ^{E}	0.578 ± 0.010 ^{E}	0.530 ± 0.031 $^{B, C}$
**L 0.5%**	0.680 ± 0.011 ^{E}	0.658 ± 0.020 ^{E}	0.588 ± 0.025 ^{E}	0.531 ± 0.024 $^{B, C}$
**O 5%**	0.006 ± 0.006 ^{A}	0.005 ± 0.009 ^{A}	0.009 ± 0.002 ^{A}	0.007 ± 0.013 ^{A}
**O 0.5%**	0.008 ± 0.013 ^{A}	0.020 ± 0.003 ^{A}	0.083 ± 0.111 ^{A}	0.376 ± 0.318 $^{B, C}$
**T 5%**	0.009 ± 0.012 ^{A}	0.002 ± 0.003 ^{A}	0.004 ± 0.007 ^{A}	0.000 ± 0.000 ^{A}
**T 0.5%**	0.334 ± 0.091 ^{C}	0.539 ± 0.020 $^{C, D}$	0.283 ± 0.064 $^{C, D}$	0.193 ± 0.120 $^{A, B}$

**TT 5%**	0.539 ± 0.094^{D}	$0.606 \pm 0.015^{D, E}$	$0.609 \pm 0.025^{D, E}$	$0.522 \pm 0.006^{B, C}$
**TT 0.5%**	$0.649 \pm 0.038^{D, E}$	0.643 ± 0.021^{E}	0.597 ± 0.017^{E}	$0.510 \pm 0.011^{B, C}$
