# Supplementary material for: The role of essential oils as eco-friendly strategy to control biofilm collected in the Colosseum (Rome, Italy)
Source: Appl Microbiol Biotechnol. 2025 Feb 18;109(1):48. doi: 10.1007/s00253-025-13433-1 (PMC11836000; doi:10.1007/s00253-025-13433-1)
Supplement: Supplementary file 1 — Supplementary file1 (PDF 233 KB) [file 253_2025_13433_MOESM1_ESM.pdf]

# The role of essential oils as eco-friendly strategy to control biofilm collected in the Colosseum (Rome, Italy)

Roberta Ranaldi[1a†*], Lorenza Rugnini[1†], Giada Migliore[2], Flavia Tasso[2], Francesco Gabriele[3], Nicoletta Spreti[3], Francesco Scuderi[1], Roberto Braglia[1], Patrick Di Martino[4], Angelica Pujia[5], Antonella Canini[1].

[1]Department of Biology, Tor Vergata University of Rome, Via della Ricerca Scientifica 1, 00133 Rome, Italy.
[a]PhD program in Evolutionary Biology and Ecology, Department of Biology, Tor Vergata University of Rome, Rome, Italy.
[2]ENEA, Department of Territorial and Production Systems Sustainability, Via Anguillarese 301, 00123 Rome, Italy.
[3]Department of Physical and Chemical Sciences, University of Aquila, Via Vetoio, Coppito, I-67100, L'Aquila, Italy.
[4]ERRMECe Laboratory, University of Cergy-Paris, rue 13 Descartes site de Neuville-sur-Oise, 95031 Cergy-Pontoise, France.
[5]Chief conservator, Parco archeologico del Colosseo, Piazza Santa Maria Nova 53, 00186 Roma
[†] These authors contributed equally to this work.
[*] Corresponding author: ranaldi.roberta@gmail.com

**Table S1.** Photosynthetic yields of treated biofilms with EOs in the study period, with initial yield value of 0.673 ± 0.019 ($t_0$). Different superscript letters in each column indicate significant differences ($p < 0.05$) in relation to the different biocides at the same time period. Data are reported as mean ± SD values.

| | Yield ($\Delta F/F_m$) | | | |
|---|---|---|---|---|
| **Treatment** | **$t_{24h}$** | **$t_{1w}$** | **$t_{1m}$** | **$t_{2m}$** |
| **CTRL + B** | $0.002 \pm 0.003^{A}$ | $0.009 \pm 0.008^{A}$ | $0.002 \pm 0.004^{A}$ | $0.000 \pm 0.000^{A}$ |
| **CTRL + E** | $0.390 \pm 0.086^{C}$ | $0.519 \pm 0.079^{B, C}$ | $0.633 \pm 0.029^{B, C}$ | $0.538 \pm 0.023^{B, C}$ |
| **CTRL -** | $0.685 \pm 0.008^{E}$ | $0.663 \pm 0.006^{E}$ | $0.575 \pm 0.006^{E}$ | $0.471 \pm 0.012^{B, C}$ |
| **B 5%** | $0.149 \pm 0.054^{B}$ | $0.440 \pm 0.037^{B}$ | $0.599 \pm 0.002^{B}$ | $0.546 \pm 0.016^{C}$ |
| **B 0.5%** | $0.662 \pm 0.015^{D, E}$ | $0.647 \pm 0.021^{E}$ | $0.600 \pm 0.018^{E}$ | $0.526 \pm 0.014^{B, C}$ |
| **C 5%** | $0.001 \pm 0.002^{A}$ | $0.003 \pm 0.004^{A}$ | $0.001 \pm 0.002^{A}$ | $0.000 \pm 0.000^{A}$ |
| **C 0.5%** | $0.010 \pm 0.008^{A}$ | $0.035 \pm 0.045^{A}$ | $0.182 \pm 0.263^{A}$ | $0.201 \pm 0.290^{A, C}$ |
| **L 5%** | $0.605 \pm 0.015^{D, E}$ | $0.627 \pm 0.013^{E}$ | $0.578 \pm 0.010^{E}$ | $0.530 \pm 0.031^{B, C}$ |
| **L 0.5%** | $0.680 \pm 0.011^{E}$ | $0.658 \pm 0.020^{E}$ | $0.588 \pm 0.025^{E}$ | $0.531 \pm 0.024^{B, C}$ |
| **O 5%** | $0.006 \pm 0.006^{A}$ | $0.005 \pm 0.009^{A}$ | $0.009 \pm 0.002^{A}$ | $0.007 \pm 0.013^{A}$ |
| **O 0.5%** | $0.008 \pm 0.013^{A}$ | $0.020 \pm 0.003^{A}$ | $0.083 \pm 0.111^{A}$ | $0.376 \pm 0.318^{B, C}$ |
| **T 5%** | $0.009 \pm 0.012^{A}$ | $0.002 \pm 0.003^{A}$ | $0.004 \pm 0.007^{A}$ | $0.000 \pm 0.000^{A}$ |
| **T 0.5%** | $0.334 \pm 0.091^{C}$ | $0.539 \pm 0.020^{C, D}$ | $0.283 \pm 0.064^{C, D}$ | $0.193 \pm 0.120^{A, B}$ |

| | | | | |
|---|---|---|---|---|
| **TT 5%** | 0.539 ± 0.094 [D] | 0.606 ± 0.015 [D, E] | 0.609 ± 0.025 [D, E] | 0.522 ± 0.006 [B, C] |
| **TT 0.5%** | 0.649 ± 0.038 [D, E] | 0.643 ± 0.021 [E] | 0.597 ± 0.017 [E] | 0.510 ± 0.011 [B, C] |
